# Supplementary material for: Robust, reproducible and quantitative analysis of thousands of proteomes by micro-flow LC–MS/MS
Source: Nat Commun. 2020 Jan 9;11:157. doi: 10.1038/s41467-019-13973-x (PMC6952431; doi:10.1038/s41467-019-13973-x)
Supplement: Supplementary file 1 — Supplementary Information [file 41467_2019_13973_MOESM1_ESM.pdf]

Supplementary Information for

Robust, reproducible and quantitative analysis of thousands of proteomes by micro-flow LC-MS/MS

Bian et al

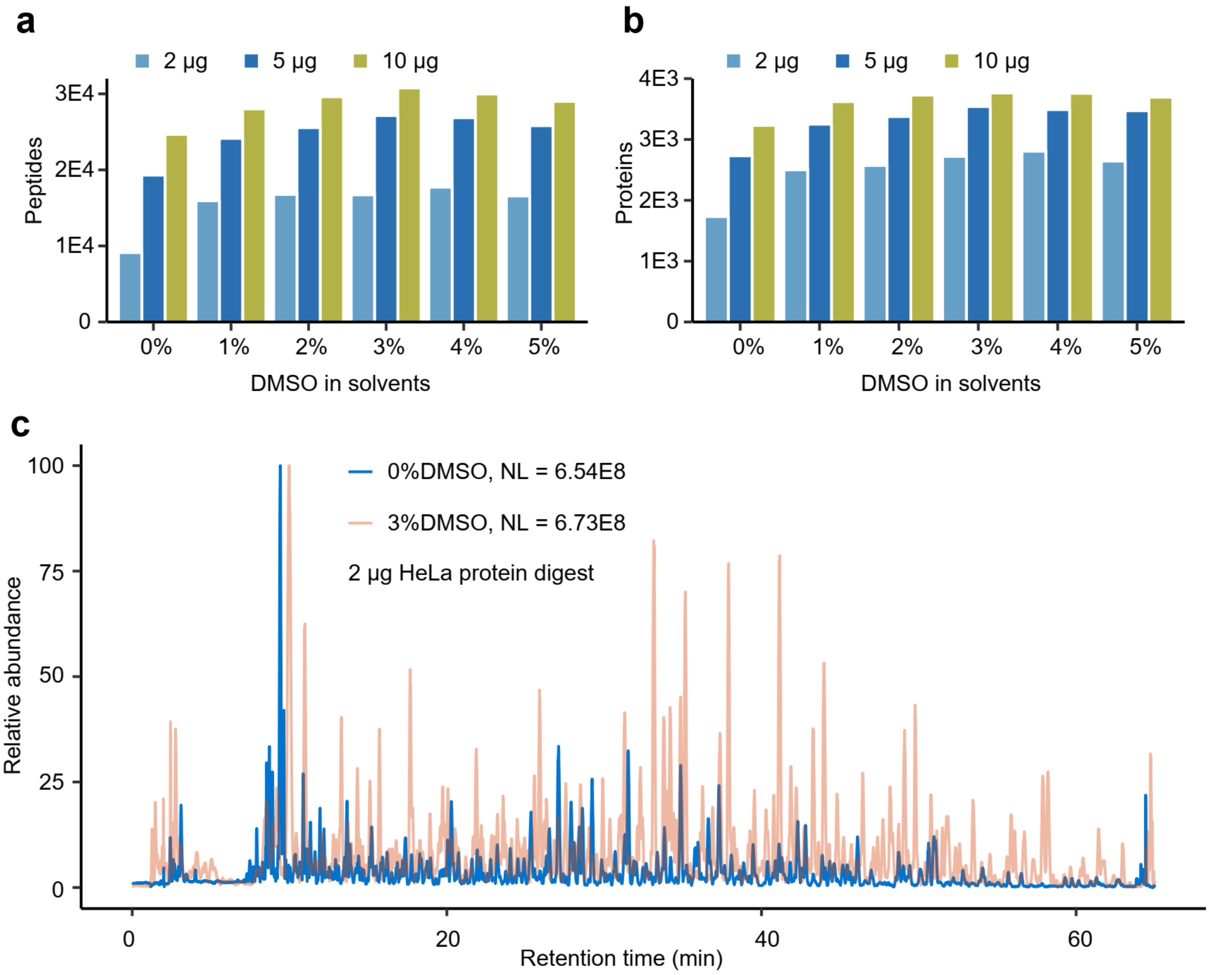

Supplementary Figure 1. DMSO improves the sensitivity of micro-flow LC-MS/MS.

(**a**) Number of unique peptide sequences identified from 2 (light blue), 5 (dark blue) and 10 µg (green) HeLa protein digests using 60 min micro-flow LC-MS/MS gradients as a function of the proportion of DMSO added to the LC solvents. Three replicate injections of 2 and 5 µg HeLa protein digest were run for each experiment, and the average peptides was used for the barplot. No replicate injection was run for the 10 µg HeLa protein digest. (**b**) Same as panel (a) but for proteins. (**c**) Overlay of base peak chromatograms of 2 µg HeLa protein digests in the absence (blue) or presence (red) of 3% DMSO in LC solvents. Source data are provided as a Source Data file for panel (**a**) and (**b**).

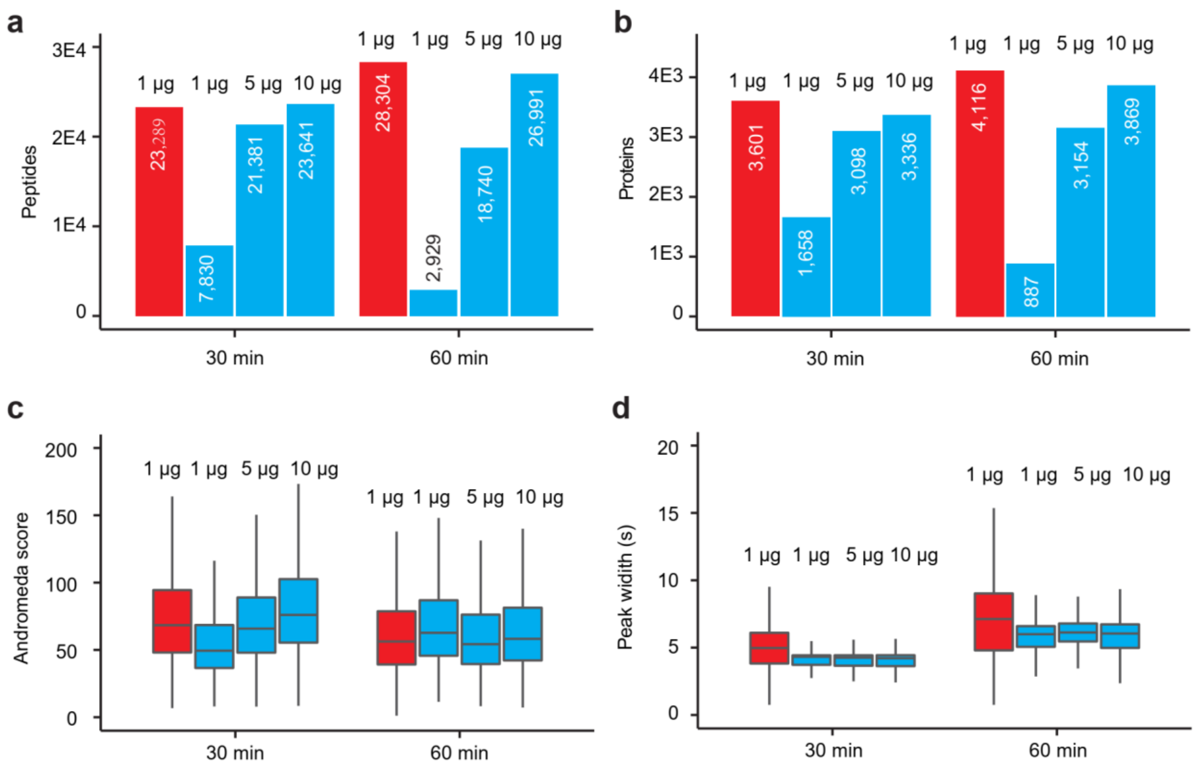

Supplementary Figure 2. Qualitative performance characteristics of the micro-flow LC-MS/MS.

The data in this figure was obtained using a Q Exactive HF-X operating an 41 Hz MS acquisition method. (**a**) Bar charts comparing peptide identifications results obtained for different sample loadings and LC gradient lengths using either nano-flow (red) or micro-flow (blue) LC-MS/MS. White numbers inside bars denote the number of peptides. (**b**) Same as panel (a) but for proteins. (**c**) Box plots comparing Andromeda peptide identification scores obtained for different sample loadings and LC gradient lengths using either nano-flow (red, n=27,476 for 30min, 33,519 for 60min) or micro-flow (blue, n=8,301 (1 µg), 24,120 (5 µg), and 26,859 (10 µg) for 30 min, and n=3,034 (1 µg), 20,437 (5 µg), and 30,373 (10 µg) for 60 min) LC-MS/MS. (**d**) Same as panel (**c**) but for peptide chromatographic peak widths. Boxes and whiskers cover 50% and 1.5 times the interquartile range of the data respectively. The numbers of peaks used for each box are 25,304 (nano), 7,816 (micro, 1 µg), 23,218 (micro, 5 µg), and 25,460 (micro, 10 µg) for 30 min, and 30,437 (nano), 1,960 (micro, 1 µg), 17,925 (micro, 5 µg), and 27,813 (micro, 10 µg) for 60 min. Source data are provided as a Source Data file for panel (**c**) and (**d**).

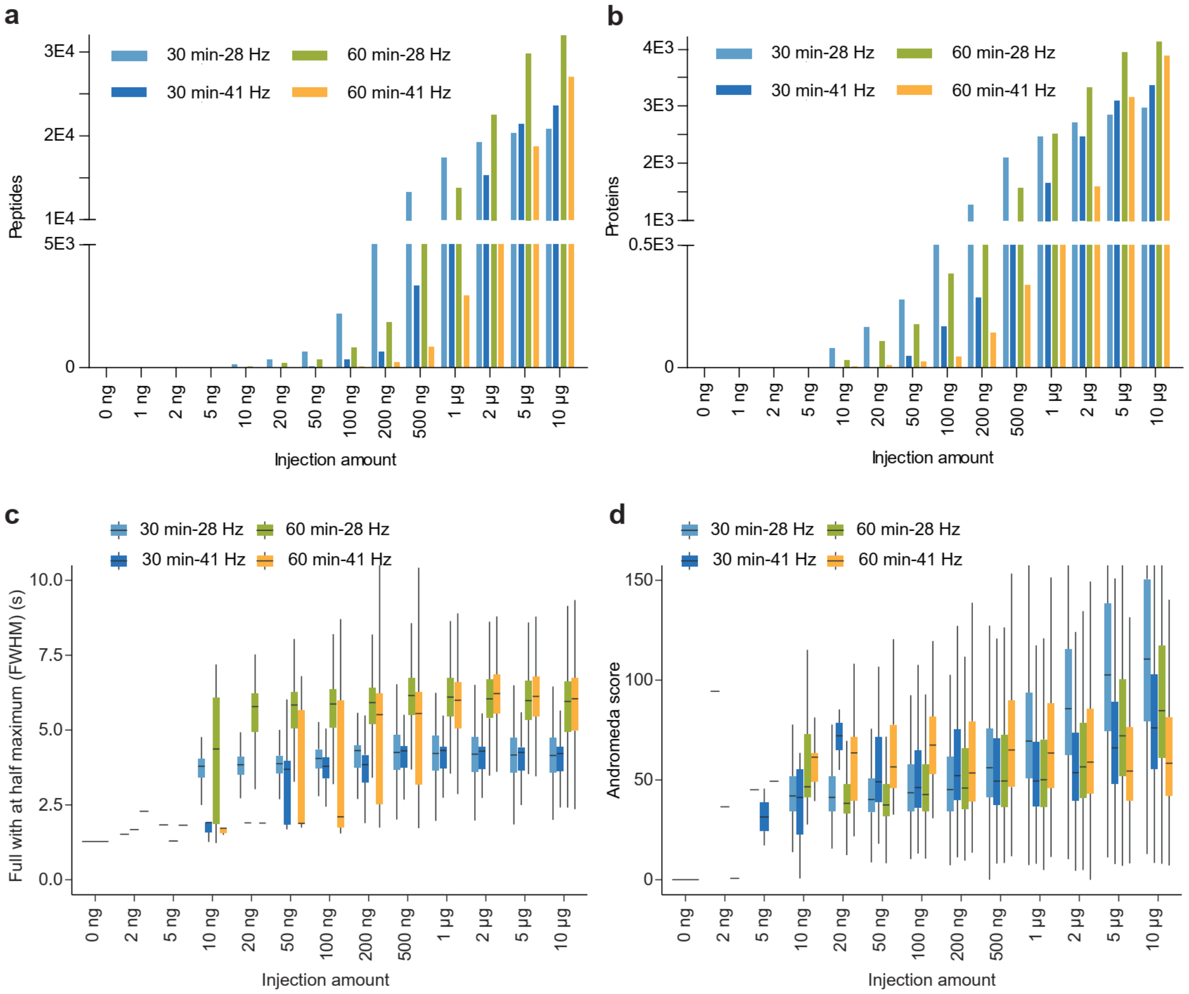

Supplementary Figure 3. Serial dilution data for HeLa protein digests.

The data in this figure was analyzed using a micro-flow LC-MS/MS system using 30 min or 60 min gradients and 28 Hz or 41 Hz MS acquisition methods on a Q Exactive HF-X. (**a**) Bar charts showing the number of identified unique peptide sequences as a function of the amount of HeLa protein digest injected. (**b**) Same as panel (a) but for proteins. (**c**) Boxplots showing the chromatographic peak width (full width at half-maximum; FWHM) distributions of peptides as a function of the amount of HeLa protein digest injected. (**d**) Same as panel (c) but for peptide Andromeda score distributions. Boxes and whiskers are defined as in Supplementary Figure 1. Note that the chromatographic peak width distributions of sample loadings of less than 1,000 ng (when analyzed by the 41 Hz MS method) or below 100 ng (when analysed by the 28 Hz MS method) are drastically different than for higher sample loadings. This apparent discrepancy arises from two issues: First, at very low sample loadings, very few peptides are identified by MS/MS (see table below) which renders the boxes and medians statistically unreliable; b) related, the 28Hz method is much more sensitive than the 41 Hz method in MS/MS mode and therefore detects more peptides. As a result, the peak widths remain more stable at lower sample quantities. MS1 parameters and MS cycle time were identical for both 28 Hz and 41 Hz methods. Source data are provided as a Source Data file for panel (**c**) and (**d**).

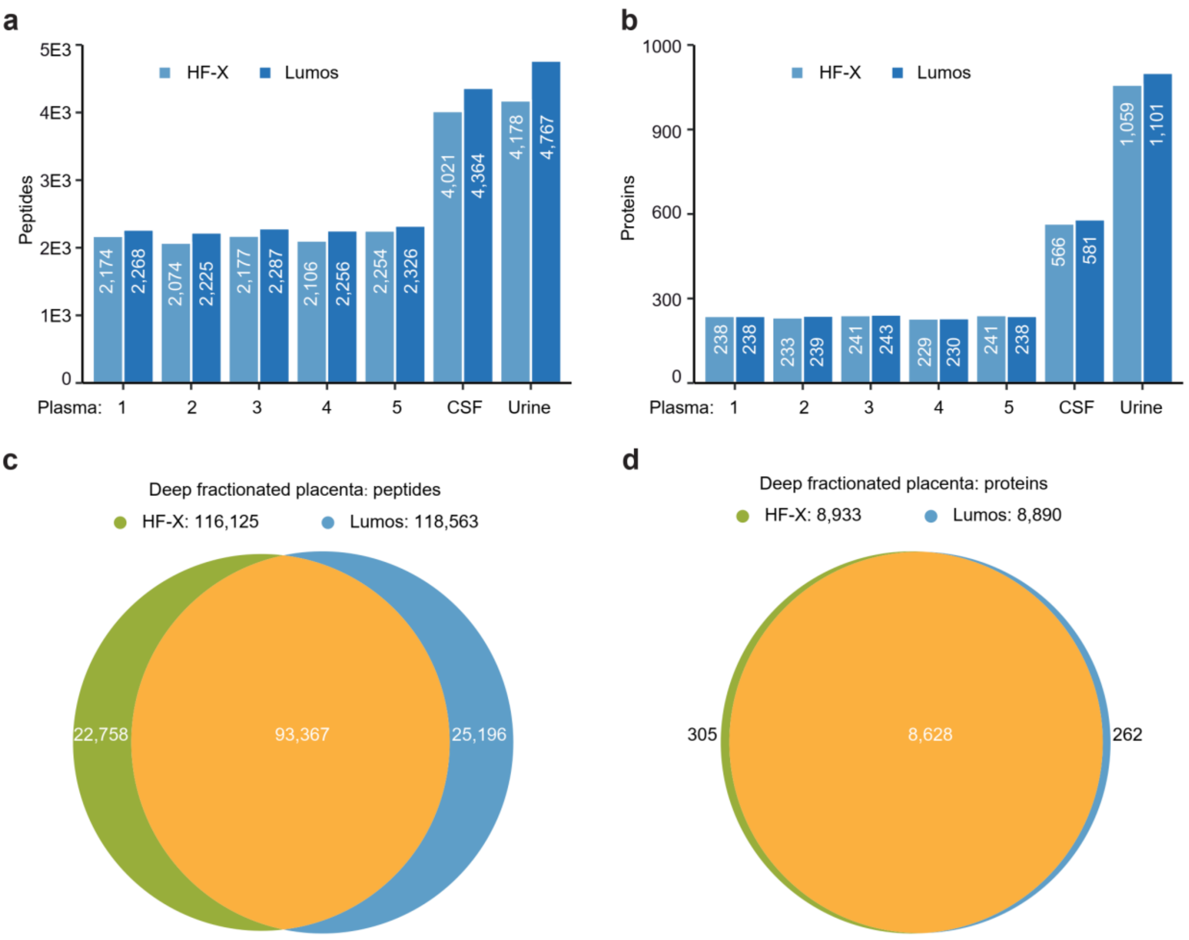

Supplementary Figure 4. Comparison of micro-flow LC-MS/MS: Q-Exactive HF-X vs. Fusion Lumos.

(**a**) Bar charts showing the number of unique peptide sequences identified from body fluid samples using micro-flow LC connected either Orbitrap Q Exactive HF-X or Orbitrap Fusion Lumos. (**b**) Same as panel (a) but for proteins. (**c**) Venn diagram comparing the total number and overlap of unique peptide sequences identified from a deep-scale analysis of a placenta protein digest (48 fractions, 15 min gradient per fraction) using micro-flow LC connected with either Orbitrap Q Exactive HF-X or Orbitrap Fusion Lumos. (**d**) Same as in panel (c) but for proteins.

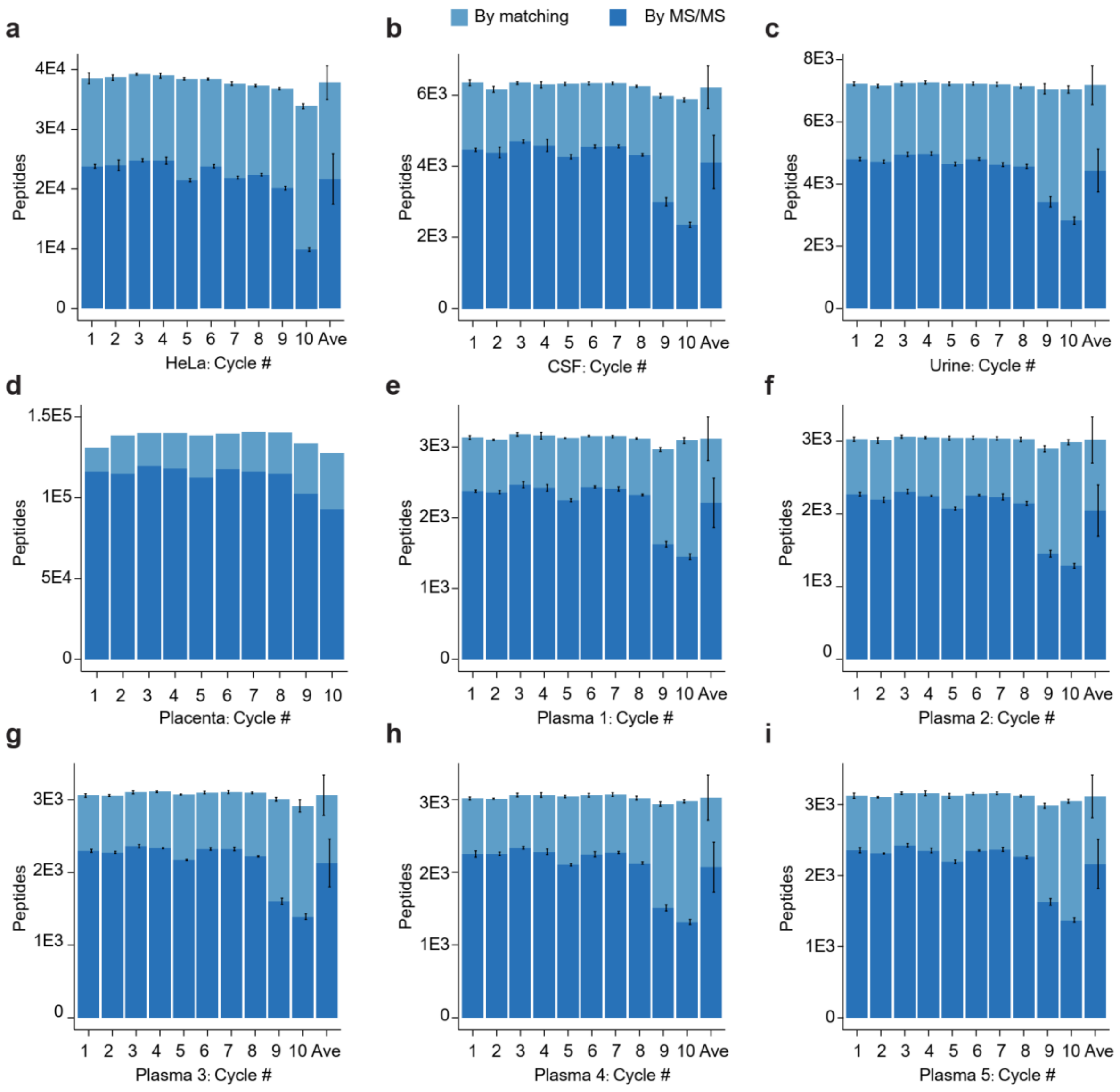

Supplementary Figure 5. Peptide identification data for the long-term performance test.

(**a-i**) Bar charts showing the number of unique peptide sequences identified by micro-flow LC-MS/MS. Bars in dark blue represent peptides identified by MS/MS and light blue represents peptides identified by including the 'match between runs' option of MaxQuant in each cycle of the 10 cycles of HeLa, CSF, urine, placenta and plasma digests from five individuals. Error bars indicate the standard deviation within each cycle, and "Ave" indicates the average number and standard deviation of all injections of this sample type in the long-term performance test, n= 20 for cycle 1 to 10, and n=200 for "Ave" bar. We note that the apparent drop in the number of peptides identified by MS/MS in cycles 9 and 10 are due to loss of sensitivity in MS/MS mode of the mass spectrometer, as most of the identifications could be recovered by matching the MS1 features. This effect is unrelated to the performance of the micro-flow LC system. Source data are provided as a Source Data file.

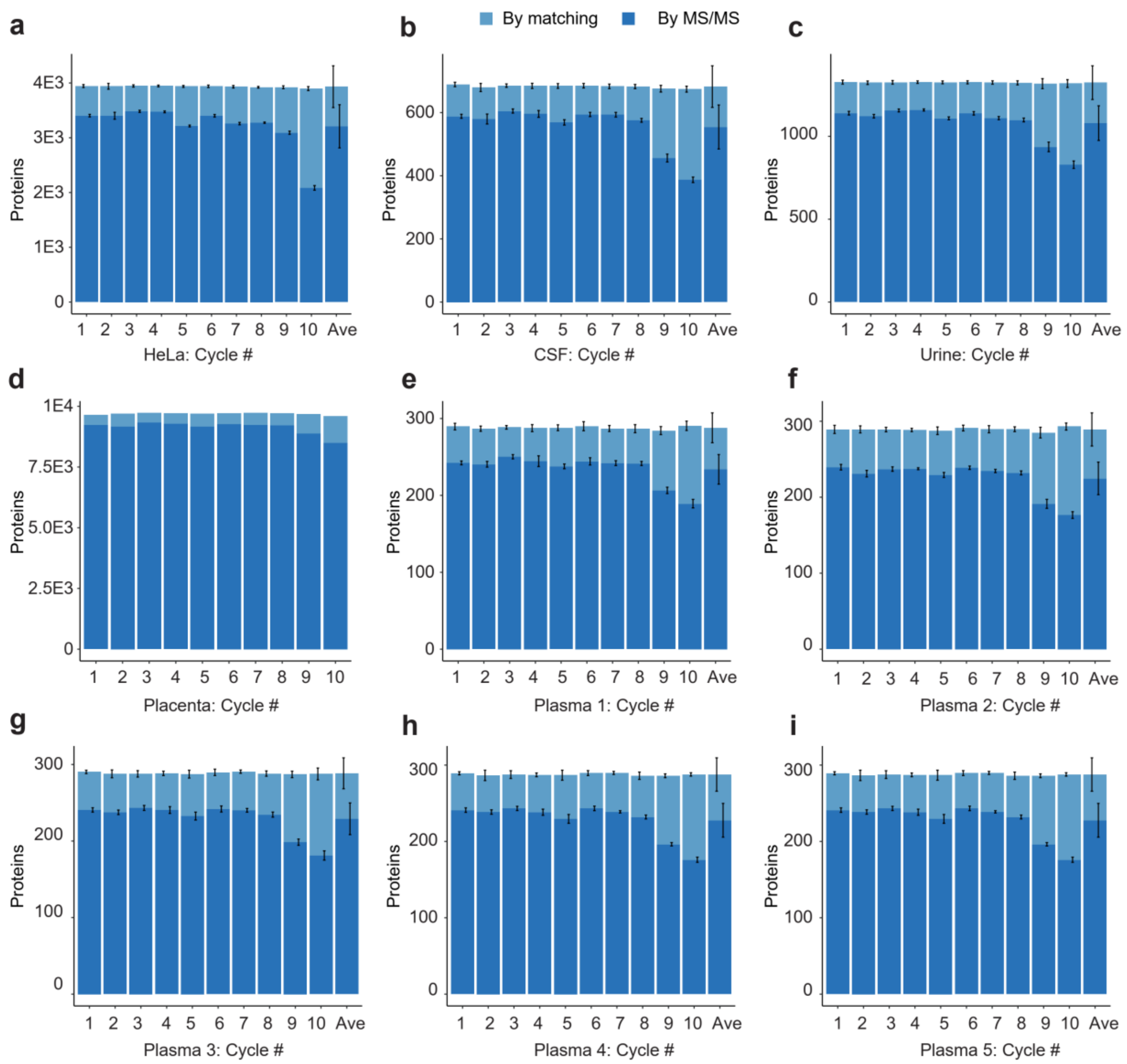

Supplementary Figure 6. Protein identification data for the long-term performance test.

(**a-i**) Bar charts showing the number of proteins identified by micro-flow LC-MS/MS. Bars in dark blue represent proteins identified by MS/MS and light blue represents proteins identified by including the 'match between runs' option of MaxQuant in each cycle of the 10 cycles of HeLa, CSF, urine, placenta and plasma digests from five individuals. Error bars indicate the standard deviation within each cycle, and "Ave" indicates the average number and standard deviation of all injections of this sample type in the long-term performance test, n= 20 for cycle 1 to 10, and n=200 for "Ave" bar. We note that the apparent drop in the number of proteins identified by MS/MS in cycles 9 and 10 are due to loss of sensitivity in MS/MS mode of the mass spectrometer, as most of the identifications could be recovered by matching the MS1 features. This effect is unrelated to the performance of the micro-flow LC system. Source data are provided as a Source Data file.

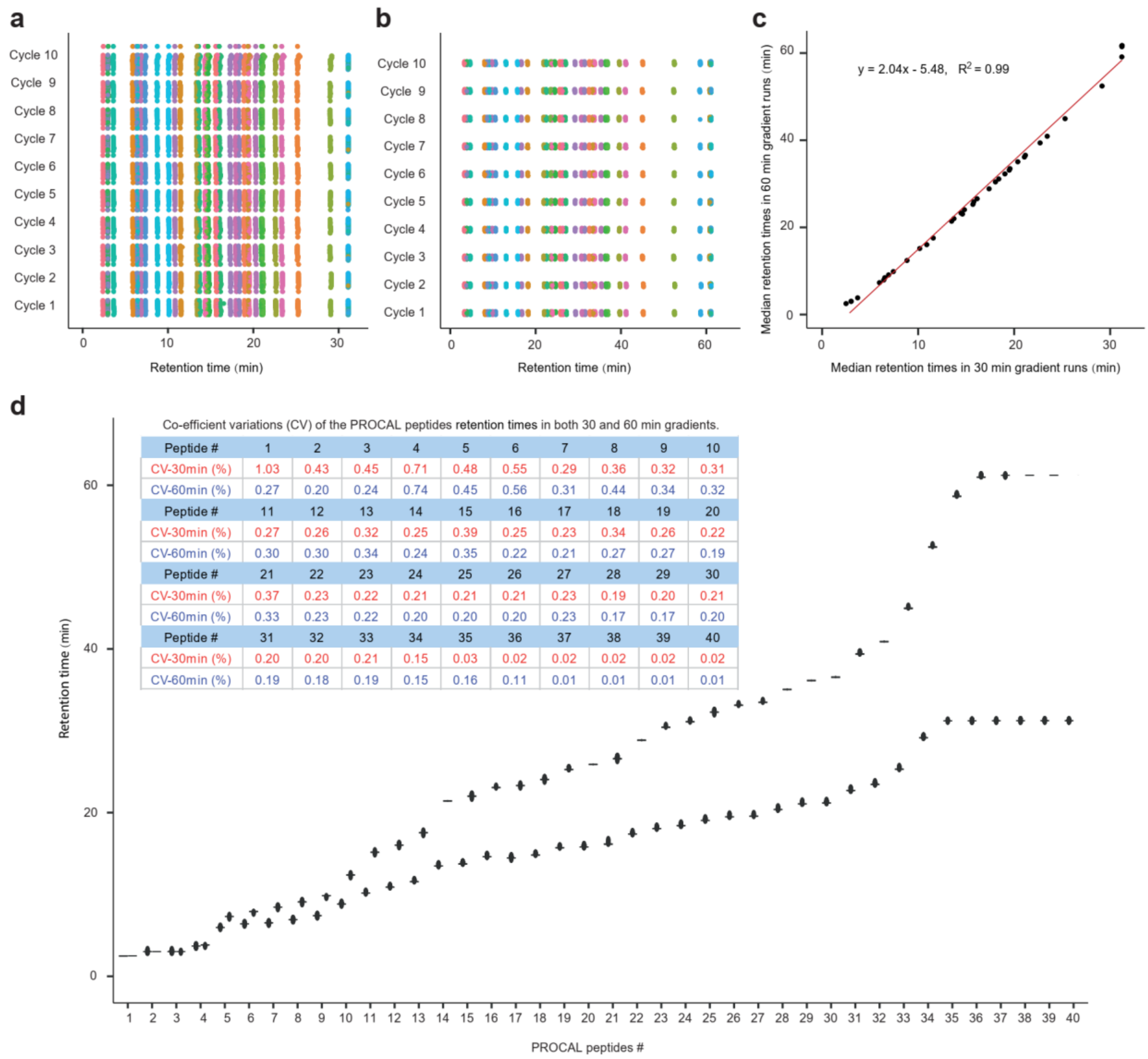

Supplementary Figure 7. Retention time reproducibility data for the long-term performance test.

(**a**) Distribution of the retention times of 40 PROCAL peptides spiked into 598 body fluid runs and 270 PROCAL only runs and analyzed using 30 min gradients (n=32,692 data points). Colors denote the different PROCAL peptides. (**b**) Same as panel (a) but for 199 injections of HeLa digests analyzed using 60 min gradients (n=7,727 data points). (**c**) Correlation analysis of median retention times of PROCAL peptides separated by 30 min or 60 min gradients. The equation describes the linear fit and R2 is the squared Pearson correlation coefficient. Source data are provided as a Source Data file. (**d**) Boxplots showing the retention time distributions of each PROCAL peptide using both 30 min (868 injections) and 60 min (199 injections) gradients. Boxes contain 50% of the data and horizontal lines indicate the median retention time. The tabular inset shows the CV values for each peptide across all injections using either 30 min or 60 min gradients. PROCAL peptides are numbered consecutively based on their retention time values.

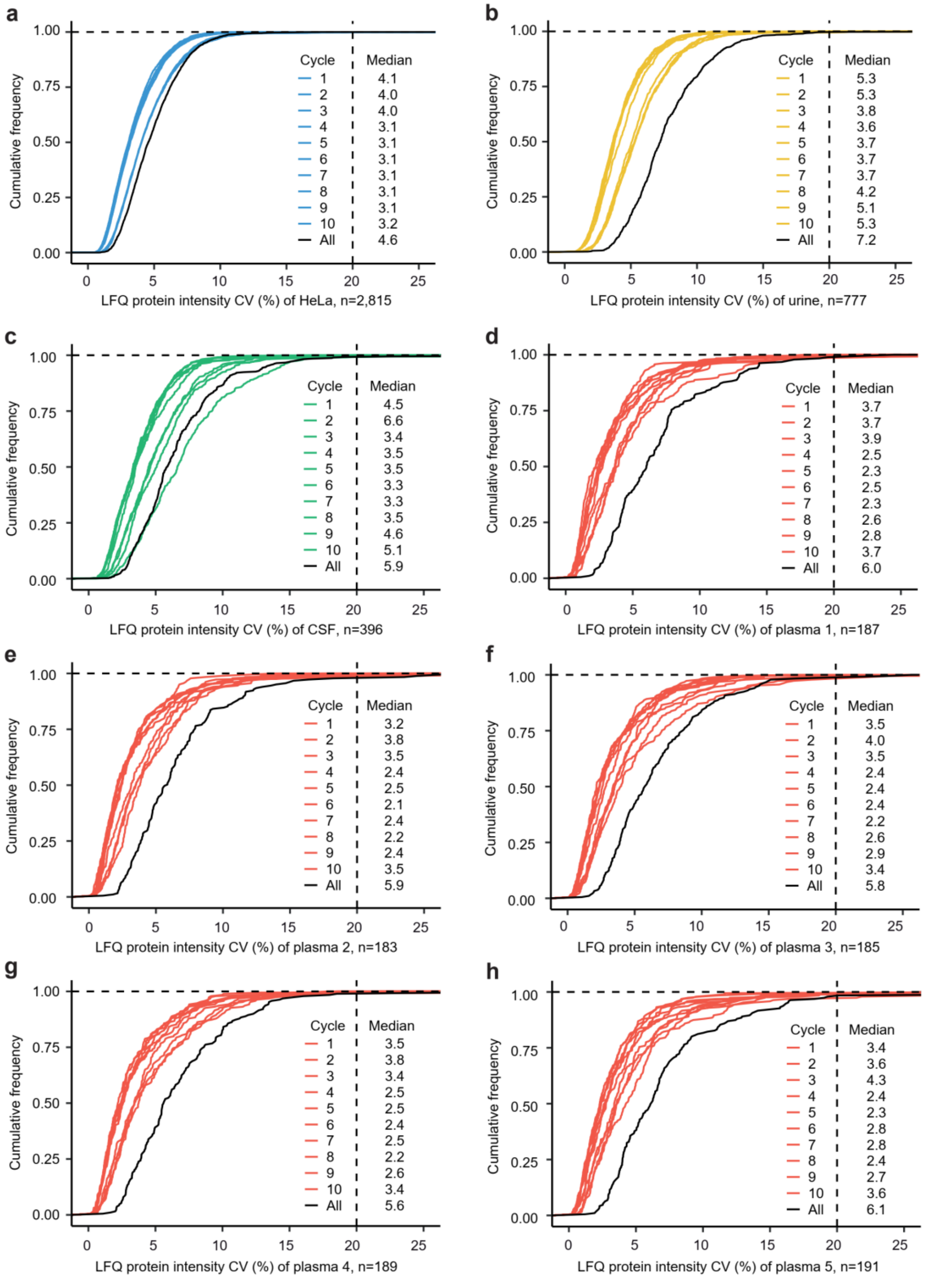

Supplementary Figure 8. Reproducibility of protein level quantification in the long-term test.

Within-cycle and across cycle quantification reproducibility of common proteins identified from 200 raw files of (**a**) HeLa digests, (**b**) urine digests, (**c**) CSF digests, and (**d-h**) 40 raw files each of plasma digests of five individuals. The across-cycle quantification reproducibility is shown as a black line in each figure and the median CV values (in %) are also given (n denotes the number of proteins identified in all samples).

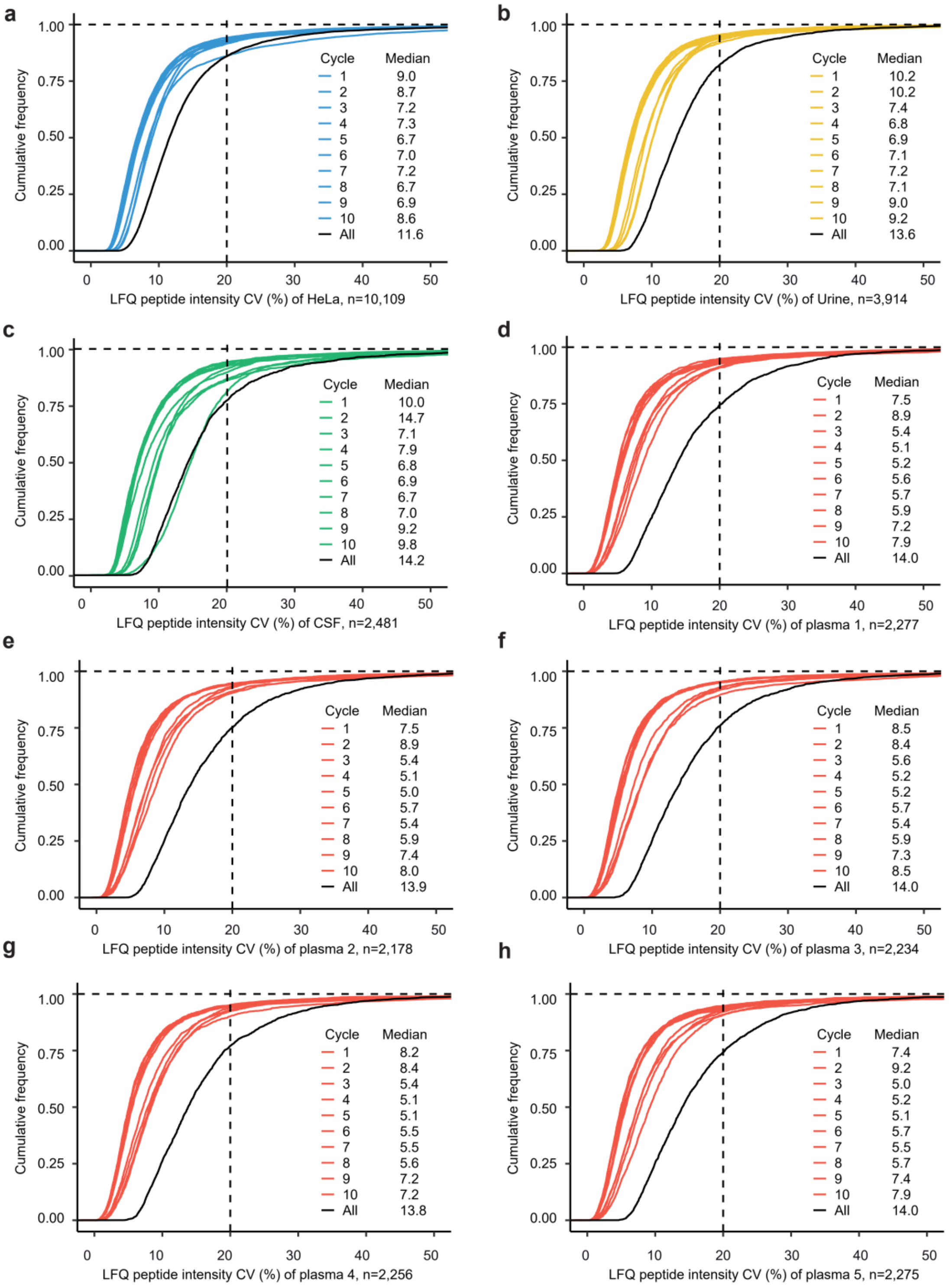

Supplementary Figure 9. Reproducibility of peptide level quantification in the long-term test.

Within-cycle and across cycle quantification reproducibility of common peptides identified from 200 raw files of (**a**) HeLa digests, (**b**) urine digests, (**c**) CSF digests, and (**d-h**) 40 raw files each of plasma digests of five individuals. The across-cycle quantification reproducibility is shown as a black line in each figure and the median CV values (in %) are also given (n denotes the number of peptides identified in all samples).

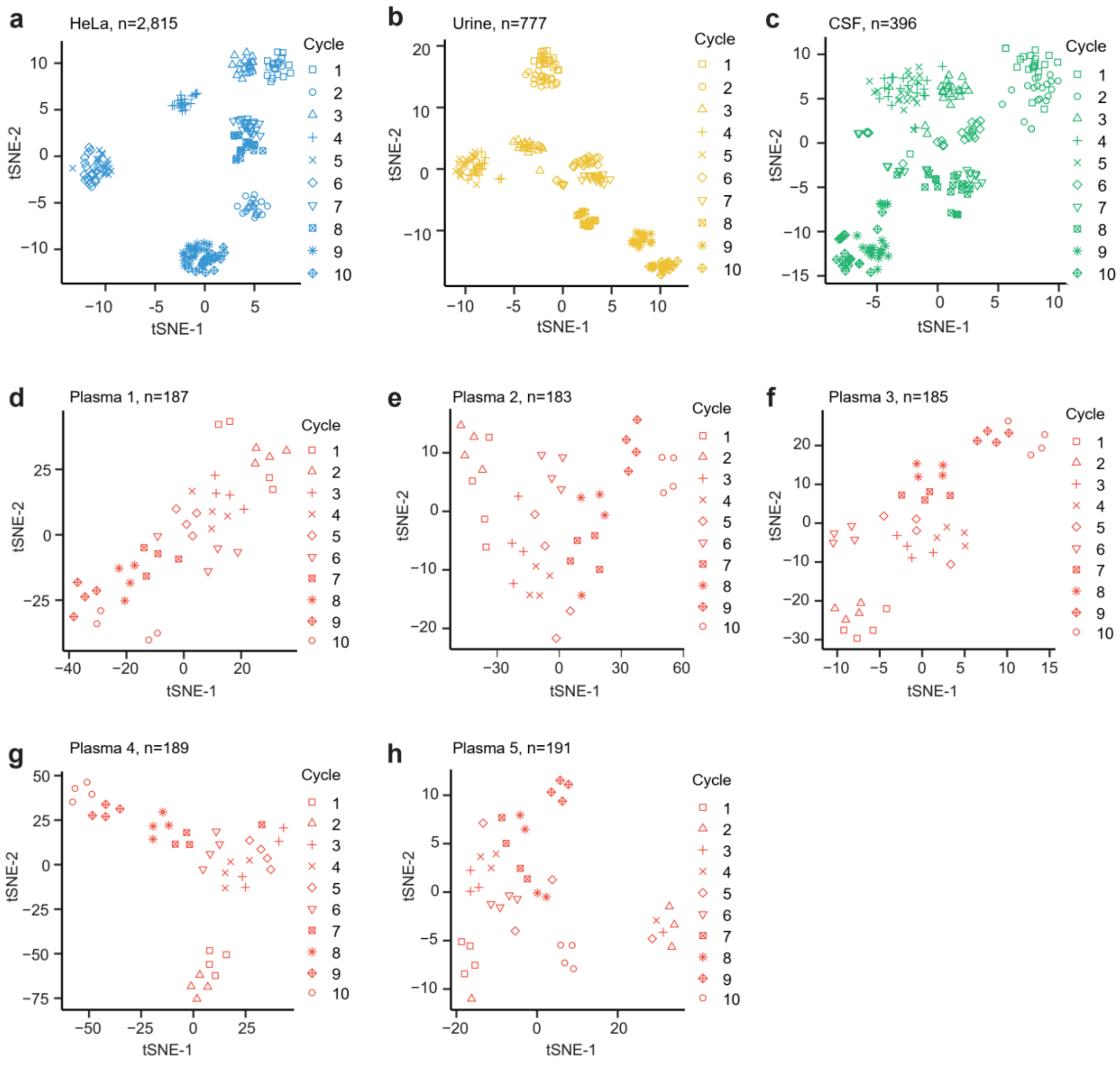

Supplementary Figure 10. Assessment of batch effects in the long-term performance test.

t-SNE analysis of 200 raw files of (**a**) HeLa digests, (**b**) urine digests, (**c**) CSF digests, and (**d-h**) 40 raw files each of plasma digests of five individuals across the 10 cycles (or batches). Batch effects were observed for some but not all sample types. We note that the t-SNE analysis brings out very small but consistent effects and should therefore not be mistaken for the presence of major batch effects. Batch effects were indeed much smaller than e. g. the person to person variability observed for the analysis of the plasma of five individuals indicating very high quantitative reproducibility of the micro-LC-MS/MS system(also see main Fig. 3c; n denotes the number of common proteins in the analysis).

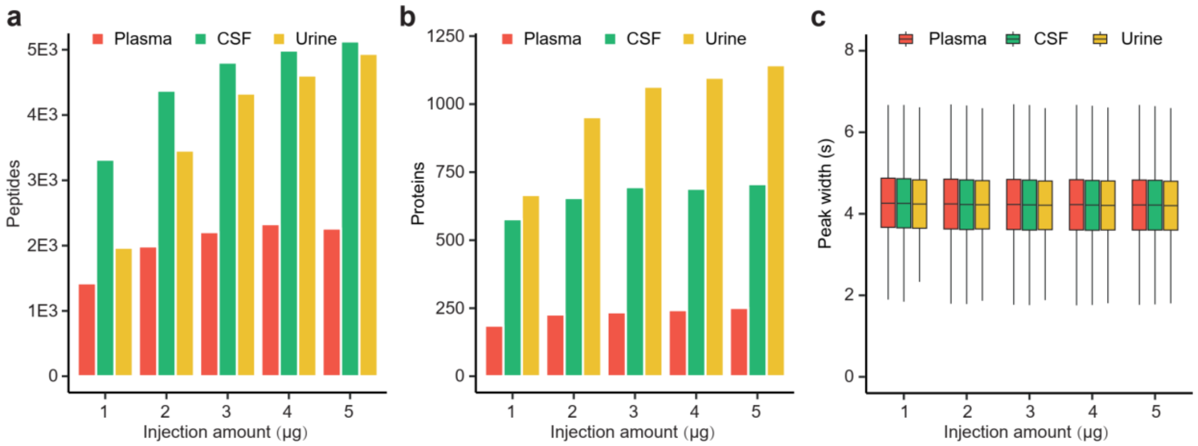

Supplementary Figure 11. Single-shot body fluid analysis by micro-flow LC-MS/MS.

(**a**) Bar charts showing the number of unique peptide sequences identified from urine, CSF and plasma protein digest as a function of the amount of sample injected. (**b**) Same as panel (a) but for proteins. (**c**) Boxplots showing the distribution of chromatographic peak widths (full peak widths at half maximum; FWHM) of all identified peptides in each experiment. The definition of boxes and whiskers is as in Supplementary Figure 2. The numbers of peaks used for each box are 1,930 (1 µg), 2,888 (2 µg), 3,288 (3 µg), 3,515 (4 µg), and 3,360 (5 µg) for plasma sample, 4,549 (1 µg), 6,215 (2 µg), 6,905 (3 µg), 7,207 (4 µg), and 7,329 (5 µg) for CSF sample, and 2,171 (1 µg), 3,940 (2 µg), 4,993 (3 µg), 5,358 (4 µg), and 5,745 (5 µg) for urine sample. Source data are provided as a Source Data file for panel (**c**).

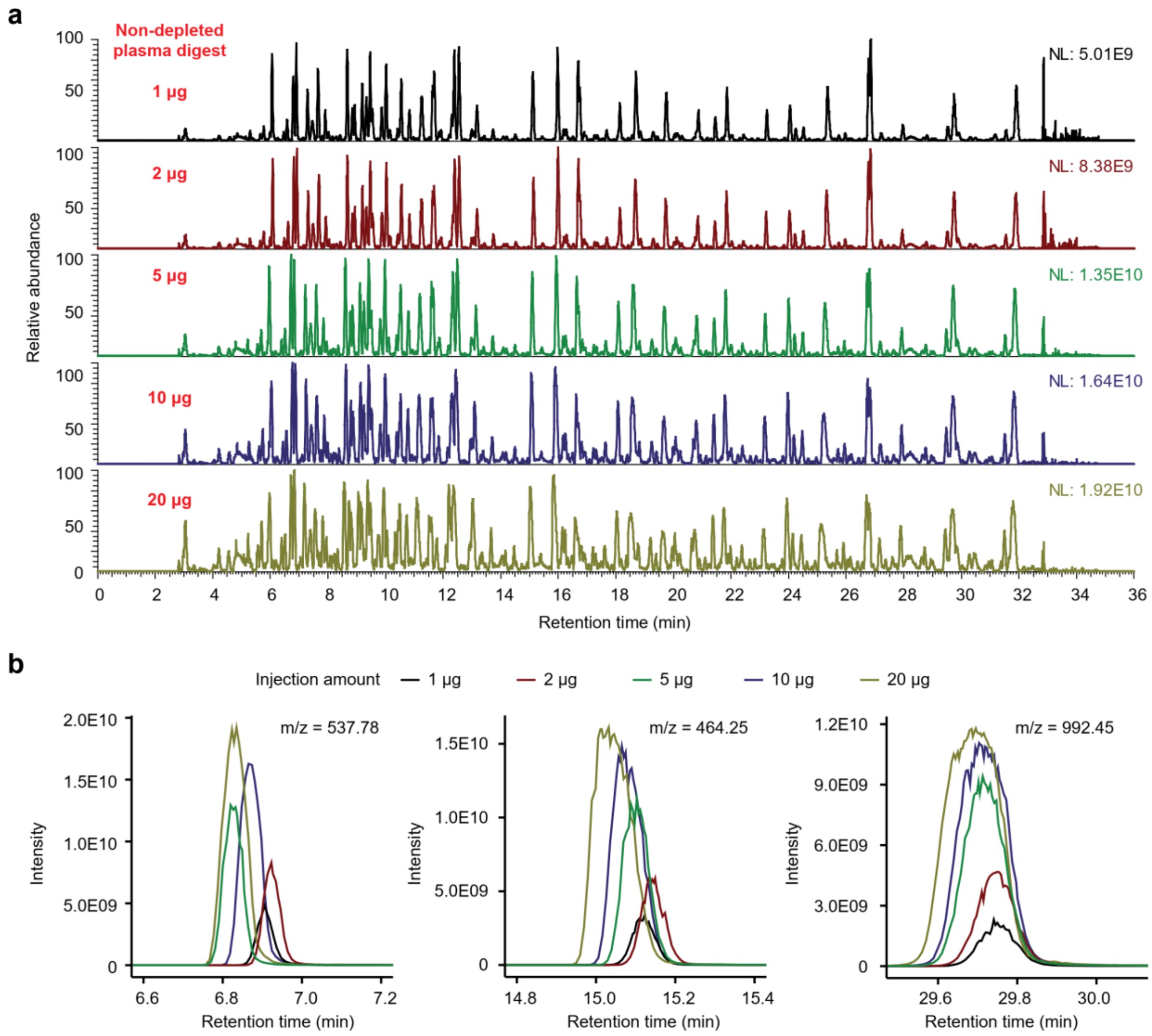

Supplementary Figure 12. Chromatographic separation performance of plasma digests.

(**a**) Base peak chromatograms of non-depleted plasma digests showing that chromatographic performance is maintained at all sample loading quantities. (**b**) Extracted ion chromatograms of three high abundant peptides from the same plasma samples. Colors denote the different injection amounts. We note that there is no obvious LC peak tailing, indicating that the column itself is not yet overloaded. We further note that the mass spectrometric signal of some peptides may saturate when injecting more than 5 µg digest.

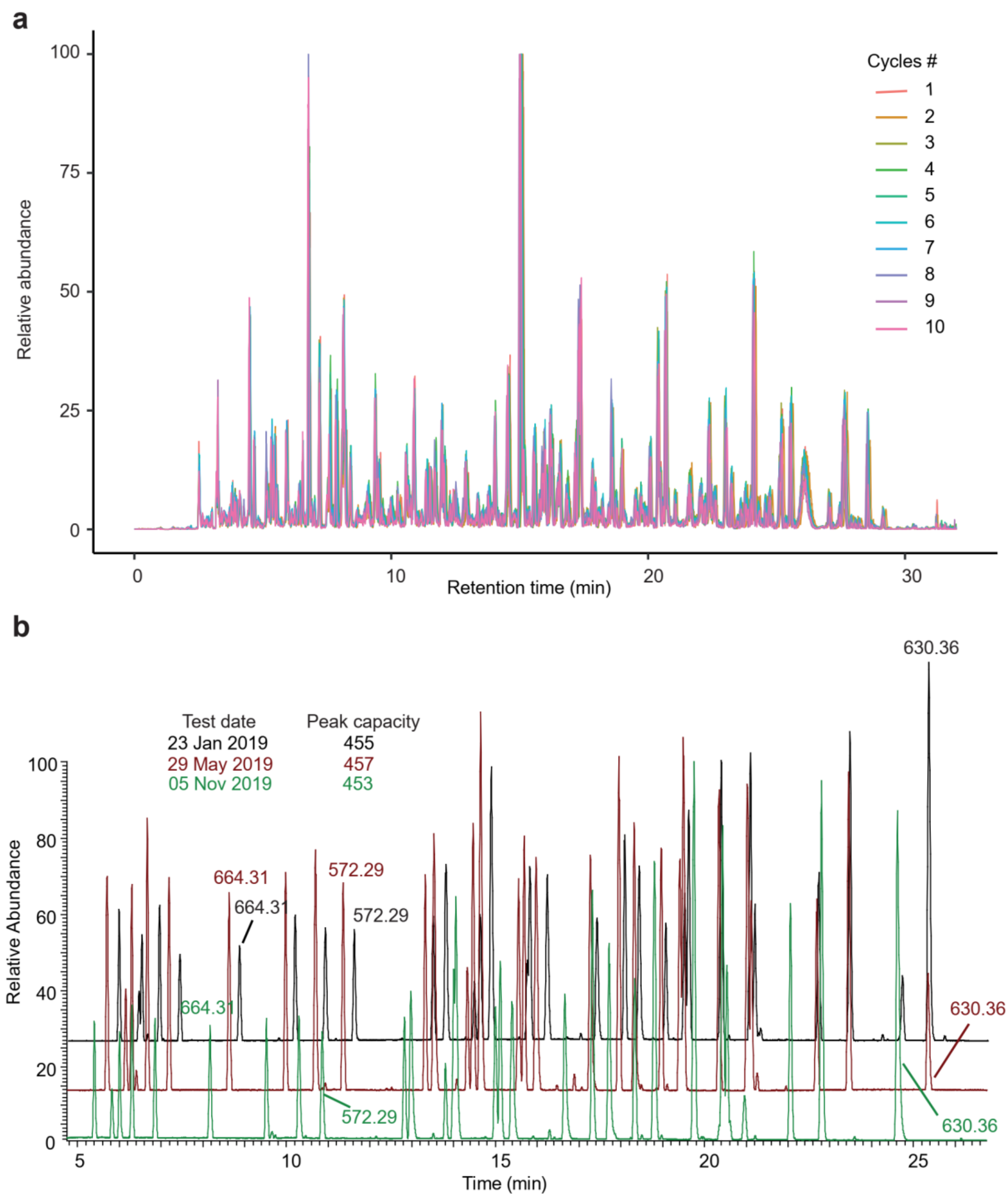

Supplementary Figure 13. Sustained separation performance across thousands of injections.

(**a**) Overlay of 10 base peak chromatograms of urine protein digests (one from each cycle of the long-term performance test, raw files were generated with approximately 4-day intervals). (**b**) Three base peak chromatograms of PROCAL peptides separated on the same column on 23 Jan 2019, 29 May 2019, and 05 Nov 2019 corresponding to injection numbers of about 1,200, 5,000 and 7,500 over the course of one year. Some local retention time shifts can be observed but the overall separation performance is maintained throughout as demonstrated by the virtually identical peak capacities calculated based on the PROCAL peptides for each separation. Source data are provided as a Source Data file for panel (**b**).

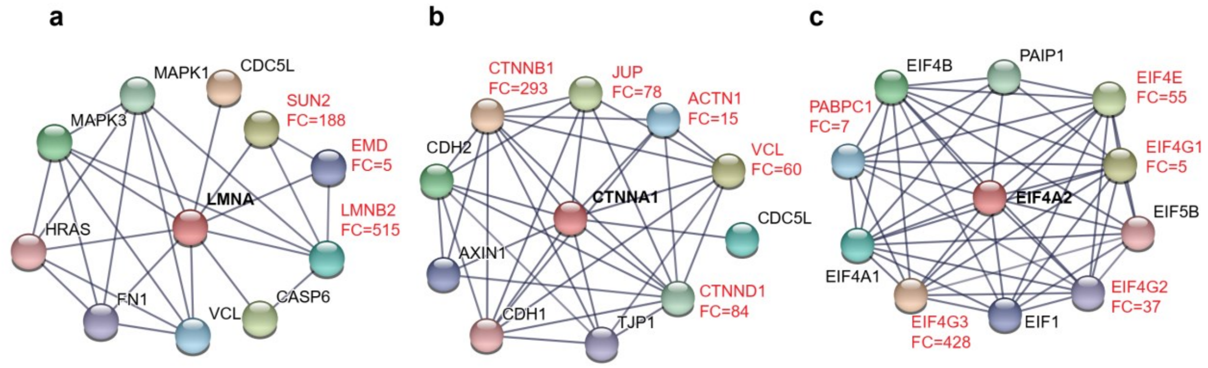

Supplementary Figure 14. STRING networks of proteins identified by BioID and AP-MS.

(**a-c**) Interaction networks based on the ten most confident interaction partners in STRING for the bait proteins LMNA, CTNNA1 and EIF4A2 respectively. The peptides generated by BioID and AP pull-downs were analyzed by micro-flow LC-MS/MS with 15 min gradient. FC denotes the fold change values of an interaction partner (over control pulldowns) assessed by SAINTexpress analysis. Proteins without FC annotations indicate that they were not identified as high confident interactors in this study.

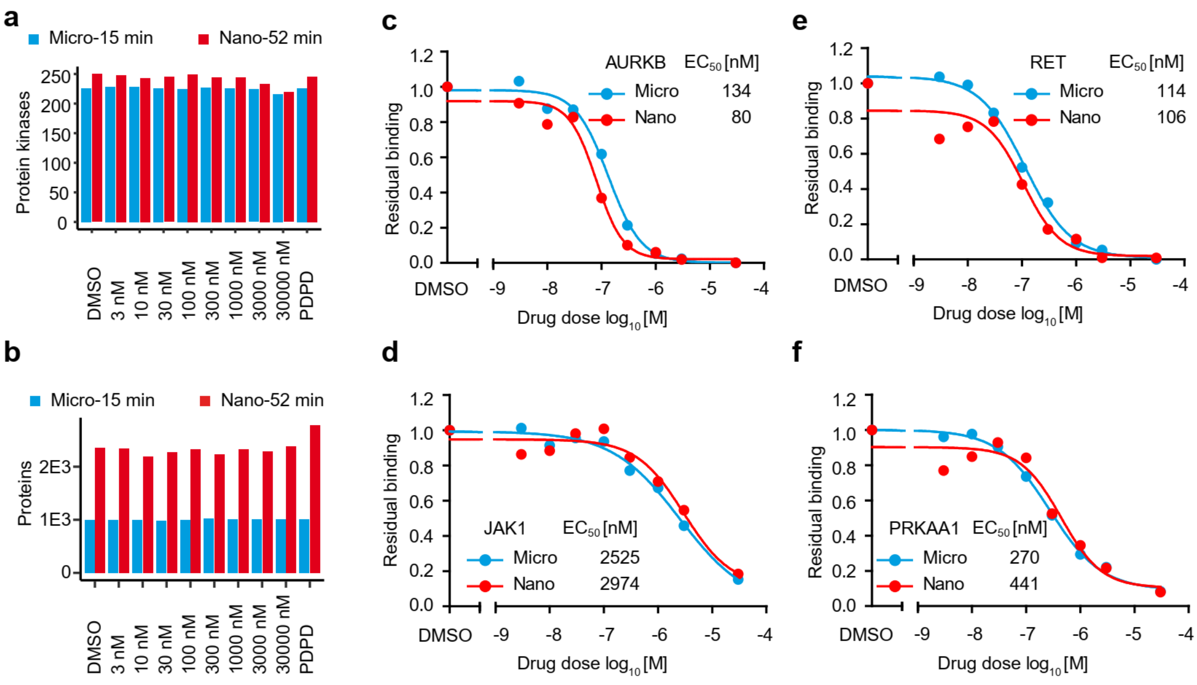

Supplementary Figure 15. Kinobeads pulldown-based target profiling of the kinase inhibitor AT-9283.

(**a**) Bar charts comparing the number of protein kinases quantified by micro-flow LC-MS/MS (15 min gradient per drug dose, Q Exactive HF-X) and nano-flow LC-MS/MS (52 min gradient per drug dose, Q Exactive HF). (**b**) Same as panel (a) but for all proteins quantified in the same pulldowns. (**c-f**) Examples for dose response curves for selected AT-9283 targets quantified by micro-flow and nano-flow LC-MS/MS, demonstrating near identical results by the two systems.

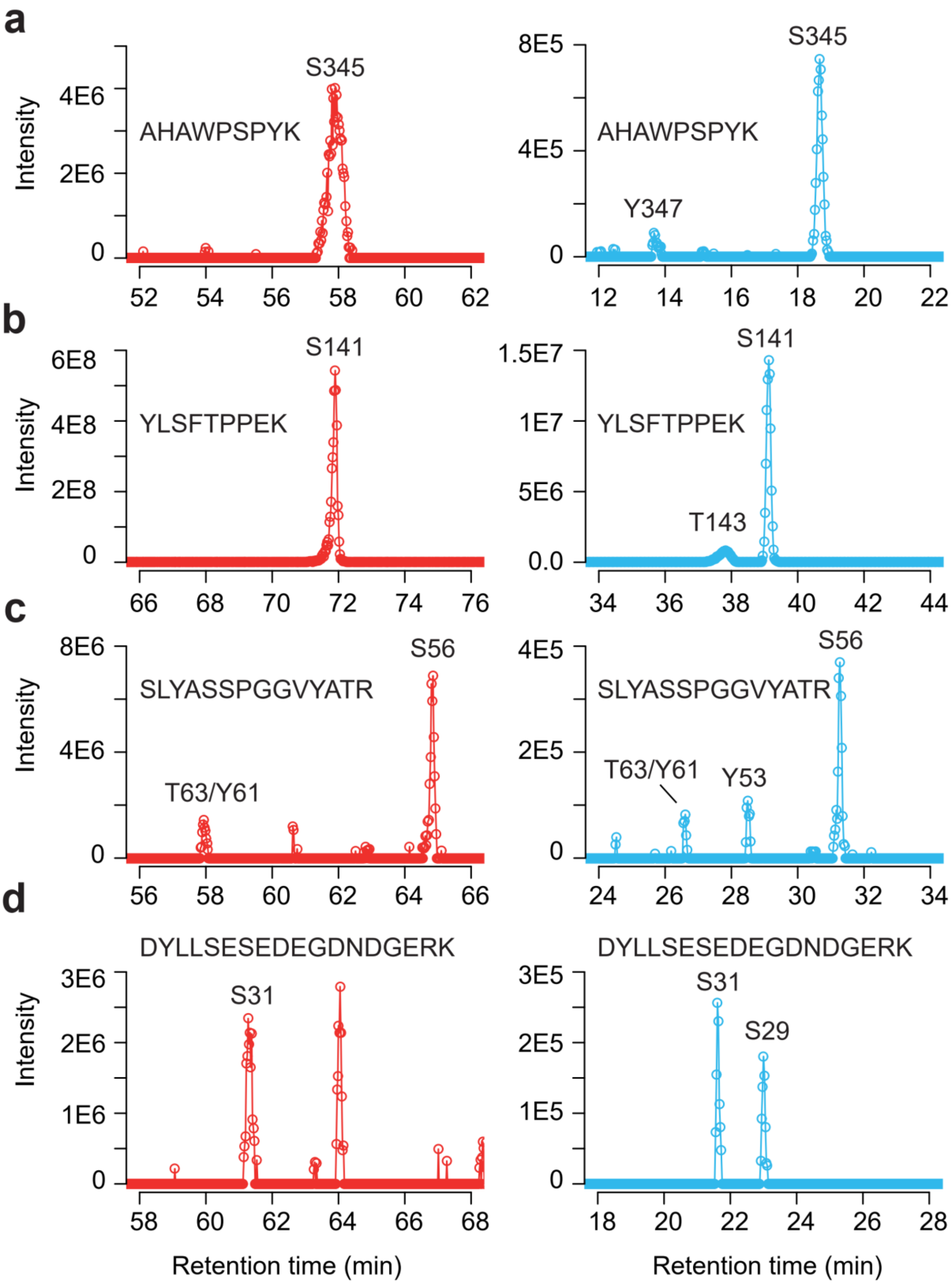

Supplementary Figure 16. Examples for the separation of phosphorylation site isomers.

(**a-d**) Extracted ion chromatograms of four peptides and their phosphorylation site isomers from an IMAC enriched HeLa protein digest and analysed by nano-flow LC-MS/MS (red) or micro-flow LC-MS/MS (blue).
