# Supplementary material for: Robust, reproducible and quantitative analysis of thousands of proteomes by micro-flow LC–MS/MS
Source: Nat Commun. 2020 Jan 9;11:157. doi: 10.1038/s41467-019-13973-x (PMC6952431; doi:10.1038/s41467-019-13973-x)
Supplement: Supplementary file 3 — Description of Additional Supplementary Files [file 41467_2019_13973_MOESM3_ESM.docx]

File Name: Supplementary Data 1.

**Description:** **(a)** Results of SAINT analysis of the BioID pulldown interactome dataset generated by the micro-flow LC-MS/MS system using 15 min gradients (Baits: NIFK, LMNA and CTNNA1). **(b)** results of SAINT analysis of the affinity purification (AP) interactome dataset generated by the micro-flow LC-MS/MS system using 15 min gradients (Baits: MEPCE and EIF4A2).

File Name: Supplementary Data 2.

**Description:** Gene names of the high confidence interactors of the five baits annotated in the previous published paper and Human Cell Map project.

File Name: Supplementary Data 3.

**Description:** Interaction network of the ten highest confidence interactors according to STRING database for the five baits.

File Name: Supplementary Data 4.

**Description:** Mapping of each plot panel in this manuscript to the original txt file uploaded to the PRIDE database and detailing the MaxQuant search results.
