# Supplementary material for: Robust, reproducible and quantitative analysis of thousands of proteomes by micro-flow LC–MS/MS
Source: Nat Commun. 2020 Jan 9;11:157. doi: 10.1038/s41467-019-13973-x (PMC6952431; doi:10.1038/s41467-019-13973-x)
Supplement: Supplementary file 8 — Reporting Summary [file 41467_2019_13973_MOESM8_ESM.pdf]

nature research

Corresponding author(s): Bernhard Kuster

Last updated by author(s): Dec 7, 2019

Reporting Summary

Nature Research wishes to improve the reproducibility of the work that we publish. This form provides structure for consistency and transparency in reporting. For further information on Nature Research policies, see Authors & Referees and the Editorial Policy Checklist .

Statistics

For all statistical analyses, confirm that the following items are present in the figure legend, table legend, main text, or Methods section.

n/a	Confirmed	
☐	☒	The exact sample size (*n*) for each experimental group/condition, given as a discrete number and unit of measurement
☐	☒	A statement on whether measurements were taken from distinct samples or whether the same sample was measured repeatedly
☒	☐	The statistical test(s) used AND whether they are one- or two-sided<br>*Only common tests should be described solely by name; describe more complex techniques in the Methods section.*
☒	☐	A description of all covariates tested
☒	☐	A description of any assumptions or corrections, such as tests of normality and adjustment for multiple comparisons
☐	☒	A full description of the statistical parameters including central tendency (e.g. means) or other basic estimates (e.g. regression coefficient) AND variation (e.g. standard deviation) or associated estimates of uncertainty (e.g. confidence intervals)
☒	☐	For null hypothesis testing, the test statistic (e.g. *F*, *t*, *r*) with confidence intervals, effect sizes, degrees of freedom and *P* value noted<br>*Give P values as exact values whenever suitable.*
☒	☐	For Bayesian analysis, information on the choice of priors and Markov chain Monte Carlo settings
☒	☐	For hierarchical and complex designs, identification of the appropriate level for tests and full reporting of outcomes
☒	☐	Estimates of effect sizes (e.g. Cohen's *d*, Pearson's *r*), indicating how they were calculated

Our web collection on statistics for biologists contains articles on many of the points above.

Software and code

Policy information about availability of computer code

Data collection	Mass spectrometry data was collected using commercial software (XCalibur, Thermo Scientific) associated with the specific mass spectrometer for each experiment.
Data analysis	1. Mass spectrometry raw files were processed with MaxQuant (version 1.6.2.3 or 1.6.0.1).<br>2. The raw files of the AP and BioID pulldown samples were also processed with Mascot and Comet, and the high confident interactions were determined using the SAINTexpress tool.<br>3. The MaxQuant output results and other data were further processed in RStudio (Version 1.1.456), most of the plots were generated with RStudio.<br>4. The GraphPad Prism 5 was used to plot the dose-response curves of kinobeads samples (Supplementary Fig. 14).<br>5. Interactome analysis was performed using online String database V11.0 (Supplementary Fig. 15).<br>6. All figures were finally organized in Adobe Illustrator CS6.

For manuscripts utilizing custom algorithms or software that are central to the research but not yet described in published literature, software must be made available to editors/reviewers. We strongly encourage code deposition in a community repository (e.g. GitHub). See the Nature Research guidelines for submitting code & software for further information.

Data

Policy information about availability of data

All manuscripts must include a data availability statement. This statement should provide the following information, where applicable:

- Accession codes, unique identifiers, or web links for publicly available datasets
- A list of figures that have associated raw data
- A description of any restrictions on data availability

All mass spectrometry files presented here have been deposited with the ProteomeXchange Consortium (http://proteomecentral.proteomexchange.org) via the PRIDE partner repository with the dataset identifier PXD015087. Reviewer account details: Username: reviewer81615@ebi.ac.uk; Password: lZw3LGt5.

Field-specific reporting

Please select the one below that is the best fit for your research. If you are not sure, read the appropriate sections before making your selection.

- [x] Life sciences
- [ ] Behavioural & social sciences
- [ ] Ecological, evolutionary & environmental sciences

For a reference copy of the document with all sections, see nature.com/documents/nr-reporting-summary-flat.pdf

Life sciences study design

All studies must disclose on these points even when the disclosure is negative.

Sample size	The overall aim of this study is to demonstrate the high reproducible, robustness and high-throughput of the high-flow LC-MS/MS system. More than 2,300 raw files were generated in this study to provide sufficient confidence to validate methodological conclusions.
Data exclusions	Three of the 1,070 raw files were excluded for the retention time analysis in the long-term performance test due to technical reasons.
Replication	1. For the DMSO effect experiment (Supplementary Fig. 1), three technical replicate injections were performed for 2 and 5 µg HeLa protein digests, and only one technical injection was performed for the 10 µg HeLa protein digest.<br>2. For the long-term performance test, all the types of single shot samples were run with multiple technical injections (200 injections of HeLa, urine, CSF samples, 270 injections of PROCAL peptides, 40 injections of each individual plasma sample, and 10 injections of deep-fractionated placenta samples).<br>3. The AP and BioID pulldown samples were performed with 2 biological replicates, and three technical replicates of pulldowns were performed for each biological replicate.<br>4. For all the other experiments in this study, we did not run duplicate technical injections for each sample.
Randomization	No randomization was applied in our sample sets.
Blinding	Blinding was not relevant to our studies.

Reporting for specific materials, systems and methods

We require information from authors about some types of materials, experimental systems and methods used in many studies. Here, indicate whether each material, system or method listed is relevant to your study. If you are not sure if a list item applies to your research, read the appropriate section before selecting a response.

Materials & experimental systems

n/a	Involved in the study	
[x]	[ ]	Antibodies
[ ]	[x]	Eukaryotic cell lines
[x]	[ ]	Palaeontology
[x]	[ ]	Animals and other organisms
[x]	[ ]	Human research participants
[x]	[ ]	Clinical data

Methods

n/a	Involved in the study	
[x]	[ ]	ChIP-seq
[x]	[ ]	Flow cytometry
[x]	[ ]	MRI-based neuroimaging

Eukaryotic cell lines

Policy information about cell lines

Cell line source(s)	1. HeLa (ATCC, CCL-2); K-562 (ATCC, CCL-243); COLO-205 (ATCC, CCL-222); MV-4-11 (ATCC, CRL-9591); SK-N-BE(2) (ATCC, CRL-2271) and OVCAR-8 (RRID: CVCL_1629).<br>2. The ten pancreatic cell lines BxPC-3, Dan-G, HPAC, HuPt-4, IMIM-PC-1, MiaPaCa2, Panc 10.05, Pa-Tu-8998-S, Pa-Tu-8998-T and PSN-1 were provided by the Dr. Med. Günter Schneider group;<br>3. HEK293 cell line was provided by the Dr. Anne-Claude Gingras group.
Authentication	1. The ten pancreatic cell lines were authenticated by Single Nucleotide Polymorphism (SNP)-Profiling conducted by Multiplexion (Multiplexion GmbH, Heidelberg, Germany) or short tandem repeat loci (STRs) profiling conducted by Microsynth (Mycrosynth, Balgach, Switzerland).<br>2. All the other cell lines have not been authenticated in the course of this study.
Mycoplasma contamination	Cell lines were tested for mycoplasma contamination in this project.
Commonly misidentified lines<br>(See ICLAC register)	No commonly misidentified cell lines were used.
